# Correction to “A combination of a dermocosmetic and a sunscreen reduces acne severity and signs of post‐inflammatory hyperpigmentation in subjects with Fitzpatrick phototypes ranging from I to VI”

**DOI:** 10.1111/jocd.16689

**Published:** 2026-03-03

Shahab R , Rashed N . J Cosmet Dermatol. 2024 Sep;23(9):3060–3061. 10.1111/jocd.16390. Epub 2024 May 30. PMID: 38813844.38813844

In the title, the word “tinted” should be deleted and photototypes range from I to VI not from IV to VI.

The title should have read “A combination of a dermocosmetic and a sunscreen reduces acne severity and signs of post‐inflammatory hyperpigmentation in subjects with Fitzpatrick phototypes ranging from I to VI”.

In paragraph 2, the used sunscreen was not tinted and no local ethic committee approval was required for the conduct of this study.

This paragraph should have read “…1785 adults with a phototype ranging from I to VI with acne and PIHP applied a with proven efficacy and a sunscreen product (DC, Effaclar Duo (+); SS, Anthelios Shaka Fluid SPF 50+, both La Roche‐Posay Laboratoire Dermatologique) daily for 2 months.^3^ The study complied with legal local requirements, and is registered in the Clinicaltrials.gov data base (NCT05601960); all subjects provided written informed consent…”.

We apologize for these errors.

Rana Shahab MD and Nabila Rashed MD, the authors.

